# Adaptation to Visual Feedback Delay Influences Visuomotor Learning

**DOI:** 10.1371/journal.pone.0037900

**Published:** 2012-05-30

**Authors:** Takuya Honda, Masaya Hirashima, Daichi Nozaki

**Affiliations:** 1 Graduate School of Education, The University of Tokyo, Bunkyo-ku, Tokyo, Japan; 2 Japan Society for the Promotion of Science, Chiyoda-ku, Tokyo, Japan; Freie Universitaet Berlin, Germany

## Abstract

Computational theory of motor control suggests that the brain continuously monitors motor commands, to predict their sensory consequences before actual sensory feedback becomes available. Such prediction error is a driving force of motor learning, and therefore appropriate associations between motor commands and delayed sensory feedback signals are crucial. Indeed, artificially introduced delays in visual feedback have been reported to degrade motor learning. However, considering our perceptual ability to causally bind our own actions with sensory feedback, demonstrated by the decrease in the perceived time delay following repeated exposure to an artificial delay, we hypothesized that such perceptual binding might alleviate deficits of motor learning associated with delayed visual feedback. Here, we evaluated this hypothesis by investigating the ability of human participants to adapt their reaching movements in response to a novel visuomotor environment with 3 visual feedback conditions—no-delay, sudden-delay, and adapted-delay. To introduce novelty into the trials, the cursor position, which originally indicated the hand position in baseline trials, was rotated around the starting position. In contrast to the no-delay condition, a 200-ms delay was artificially introduced between the cursor and hand positions during the presence of visual rotation (sudden-delay condition), or before the application of visual rotation (adapted-delay condition). We compared the learning rate (representing how the movement error modifies the movement direction in the subsequent trial) between the 3 conditions. In comparison with the no-delay condition, the learning rate was significantly degraded for the sudden-delay condition. However, this degradation was significantly alleviated by prior exposure to the delay (adapted-delay condition). Our data indicate the importance of appropriate temporal associations between motor commands and sensory feedback in visuomotor learning. Moreover, they suggest that the brain is able to account for such temporal associations in a flexible manner.

## Introduction

Appropriate associations between motor commands and their sensory consequences are important for motor learning. One of the challenges faced by the central nervous system (CNS) in accomplishing this association is the feedback delay inherent in the sensorimotor loop. Physiological studies have shown that the cerebellum is equipped with a mechanism that compensates for this time delay [1]. Cerebellar long-term depression (LTD), which is one of the cellular bases of learning, is maximally induced when climbing fiber signals (i.e., sensory errors) are delayed by approximately 250 ms with respect to the parallel-fiber signals (i.e., motor commands) [2]. In accordance with this finding, a behavioral study showed that the rate of prism adaptation decreased in line with an artificially introduced increase in visual feedback delay [3]. These findings suggest that physical feedback delay in the sensorimotor loop is a crucial parameter in motor learning.

Given our perceptual ability to associate our own actions with their sensory consequences, it is possible that the CNS can overcome the negative effects of artificially introduced feedback delays on motor learning. Recent psychophysical studies have demonstrated that the perceived time between a voluntary action and its sensory consequence is not fixed, but modifiable [4], [5]. When human participants were repeatedly exposed to an artificially introduced 250-ms delay between a key press and its consequent tone, the perceived time delay was decreased by approximately 100 ms. Specifically, intentional actions (i.e., a key press) are perceived as shifted forward in time towards their sensory consequences (i.e., a tone), while sensory consequences are perceived as shifted backwards in time towards their intentional actions. Such perceptual shifts have frequently been observed in auditory, visual, and tactile feedback tasks [6]–[8]. They are considered to reflect the causal binding between actions and their sensory consequences, to produce a coherent experience of our own actions.

Importantly, such perceptual binding is compatible with a recent theoretical framework of motor control, which involves a predictive model called the forward model [9]–[11]. In this framework, the efference copy of a motor command is processed to predict its sensory consequence, before actual sensory feedback is available. The motor control system uses this sensory prediction to correct the ongoing movement, without depending on delayed sensory feedback [12]–[14]. Additionally, it combines the prediction with actual sensory feedback [15]–[18], resulting in a reliable estimation of current movement states [19]. Accurate predictions from the forward model are crucial for fast and accurate movements. Thus, the brain evaluates the accuracy of the prediction by comparing it with the actual sensory feedback, and by modifying the forward model according to the prediction error [20]–[22].

Perceptual binding occurs only when there are voluntary motor commands, and a reliable temporal relation between action and a sensory event [4], [5]. Thus, perceptual binding is believed to result from recalibration of the feedback delay between motor commands and their sensory consequences in the predictive motor control process. We hypothesized that, if appropriate temporal associations between motor commands and their sensory consequences are created in the brain by the recalibration process, these associations may alleviate deficits of motor learning associated with delayed visual feedback.

To test this hypothesis, we examined the ability of human participants to adapt their reaching movements to a novel visuomotor environment with or without the presence of a delay between a movement and its visual feedback. A previous study [23], using a prism adaptation paradigm, examined the way in which repeated exposures to a delay in visual feedback influenced the subsequent prism adaptation during reaching. However, it failed to demonstrate a beneficial effect of the exposure. This may be attributed to 2 factors. Firstly, the study displayed only the endpoint error as visual feedback, and the participants did not see the entire hand path. Given the importance of continuous visual feedback for motor control and learning [18], [24], [25], it is possible that the beneficial effect of repeated delay exposure in motor learning is observed only when providing continuous visual feedback. Secondly, the sudden application of a prism perturbation made the participants aware of the discrepancy between the hand and target. This allowed them to engage some strategic processes during adaptation [26], which could influence the implicit motor learning process. Thus, it is important to ensure that the participants are not aware of the introduced perturbation. In the present study, we introduced an artificial delay to a cursor that was continuously visible, and gradually increased the amount of visual rotation so that the participants were unaware of its presence.

## Methods

### Ethics Statement

This study was conducted according to the Declaration of Helsinki. The experimental procedures were approved by the ethics committee of the Graduate School of Education at the University of Tokyo. Written informed consent was obtained from all participants prior to the experiments.

### Participants

Thirty-six volunteers (28 men and 8 women; age range, 19–28 years) participated in this study. Participants were randomly assigned to 1 of 3 experimental conditions, i.e., each group had 12 participants, and each participant completed only 1 experimental condition. Participants had no cognitive or motor disorders, and were naïve to the visuomotor adaptation task and to the purpose of the experiment. Their dominant hands were determined by the Edinburgh Handedness Inventory [27]; all participants were right-handed. They were financially compensated for their time.

### Apparatus

**Figure 1:**
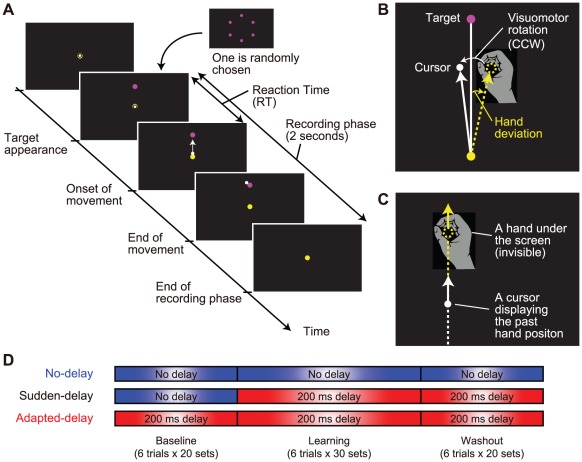
Experimental setting. (**A**) The sequence of events in a trial. (**B**) As a visuomotor learning task, we used a visuomotor rotation in which the direction of the cursor was rotated from the direction of the hand around the starting position. (**C**) In the delayed cursor experiment, the cursor represented the hand position that had occurred 200 ms previously. (**D**) Three experimental conditions for the cursor display. In the no-delay condition, there was no artificial delay (cyan box) between the hand and cursor throughout the experiment. In the sudden-delay condition, a 200 ms delay was artificially introduced (magenta box) during the learning and washout sessions. In the adapted-delay condition, a 200 ms delay was artificially introduced (magenta box) at the start of the baseline session and maintained throughout the experiment.

Participants sat on a straight-backed chair, while grasping the handle of a robotic manipulandum with their right hand (Phantom Premium 1.5HF, SensAble Technologies, Wilmington, MA, USA). A spring simulated by the device (1.0 N/mm) generated a virtual horizontal plane, on which the handle movement was restricted. A projector was used to display the position of the handle by means of a cursor (8-mm diameter white circle) on a horizontal screen (45 cm×60 cm), placed 13 cm above the virtual plane and 10–15 cm below shoulder level. Thus, the screen board prevented the participants from directly seeing their arm and the handle. Participants controlled the cursor from a start position (10-mm diameter yellow circle) to a target (10-mm diameter magenta circle), which were also displayed on the screen (Fig. 1A). After completion of the reach, the device automatically returned the hand and handle to the start position, by applying a spring-like force toward the start position. During this automatic movement, the cursor was extinguished from the screen. Therefore, the participants could concentrate only on the reach toward the target in each trial. The start position was located approximately 25 cm in front of the body, and the distance between the start position and the target was 9 cm. In each trial, the target was randomly chosen from 6 equally spaced positions on a circumference. The start point was always visible. The position and velocity of the handle were recorded with a sampling frequency of 500 Hz for offline analysis.

### Procedures

#### Instructions

Participants were instructed to move the cursor from the start position to the target with a straight, fast, and uncorrected stroke [21], [28], and to initiate the reaching movement as soon as the target was presented. The uncorrected stroke was requested to eliminate any effect of possible differences in online movement correction between the 3 experimental conditions, on motor adaptation performance. Participants were instructed to maintain the hand position where it stopped after each stroke, and not to correct this position, even if the cursor was not on the target.

#### General procedure

We arranged a series of 6 different target trials into 1 set. Within each set, the order of the 6 target trials was randomized. The entire experiment consisted of 70 sets (420 trials), and lasted for approximately 40 minutes. All participants performed the experiment without a rest break. The sets were organized into 3 sessions: 20-sets baseline session, 30-sets learning session, and 20-sets washout session. In the baseline and washout sessions, the direction of the cursor movement was the same as that of the hand movement. In the learning session, the direction of the cursor movement was rotated around the start position, counterclockwise from the direction of the hand movement (Fig. 1B). During sets 21–40 (i.e., the first 20 sets in the learning session), this visual rotation angle was gradually increased from 0° to 20°. After the rotation angle reached 20°, it was kept constant for 10 sets until the end of the learning session. Aside from the visuomotor rotation, we inserted a 200-ms delay between the cursor and the hand position, i.e., the position of the cursor displayed the hand position that had occurred 200 ms previously (Fig. 1C). Once the delay was inserted, it was not removed until the end of the experiment. The timing of the insertion differed according to the experimental conditions (Fig. 1D).

#### Conditions

Figure 1D shows the time delay between the actual hand position (invisible) and the cursor position (visible) for the following 3 conditions: (1) the no-delay condition, in which the cursor moved synchronously with the hand throughout the experiment; (2) the sudden-delay condition, in which a 200-ms delay was artificially introduced between the hand position and the cursor position (Fig. 1C) during the learning and washout sessions; and (3) the adapted-delay condition, in which the 200-ms delay was introduced at the start of the baseline session and maintained throughout the experiment; this allowed the participants to become accustomed to the delay before encountering the visual rotation. By comparing the 3 conditions, we were able to evaluate whether motor adaptation was affected either by the delay (no-delay vs. sudden-delay), or by exposure to the delay in baseline sessions (sudden-delay vs. adapted-delay).

It should be noted that, even in the no-delay condition, there was a physical delay between the handle movement and cursor position movement, because of the data processing time of the computer. To measure this delay, the handle and cursor positions were recorded by a high-speed video camera (EX-F1, Casio, Japan) with a sampling frequency of 1200 Hz, while the handle was moved randomly back and forth in a virtual one-dimensional channel. Cross-correlations were calculated between the handle and cursor velocities, and the lag at which the correlation was highest was determined as the physical delay. This physical delay was found to be 60 ms. Nevertheless, for clarity, we hereafter refer to this condition as the no-delay condition, in the sense that there is no delay other than the experimentally unavoidable delay. With respect to the 200-ms delay for the sudden-delay and adapted-delay conditions, the measured physical delay was 259.2 ms, indicating that the additional 200-ms delay was appropriately controlled.

### Data analysis

The handle position data were low-pass filtered using a zero-lag fourth-order Butterworth filter (cutoff frequency, 5 Hz). The hand position at peak velocity (PV) was used to calculate the movement direction of the hand for each trial (Fig. 1B). The clockwise deviation of the hand direction from the target was defined as positive, which reflected visuomotor adaptation (Fig. 1B). The mean value of the deviations for the 6 target trials in each set was used as a measure of the degree of adaptation. The learning index was defined as the average level of adaptation evident through sets 41–50, where the visuomotor rotation was maintained at 20°. When the participants did not change the movement direction of the hand regardless of visual rotation, the learning index would be 0°. By contrast, if they adjusted their reaching direction clockwise so that the cursor reached the target correctly, the learning index would be close to 20°.

We also calculated the learning rate based on a state-space model:
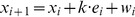
(1)

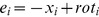
(2)where 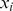 represents the internal state of the system indicating the hand direction at the 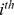 set; 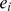 represents the cursor error observed at the 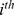 set; 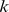 is a learning rate, representing how the internal state is updated according to error information; 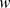 represents Gaussian white noise with a mean of 0 and standard deviation of 1; and 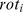 is an imposed visual rotation at the 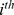 set. To compare the present result directly with previously reported results, we did not consider the spontaneous memory loss (i.e., the constant coefficient before the term 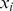 is assumed to be 1; see Kitazawa et al. [3]). Although the learning rate could change across trials (e.g., if the participants gradually adapted to the sudden-delay condition), for simplicity, we assumed that it remained constant throughout the learning and washout sessions. Further, in order to make direct comparisons with previous studies [3], [23], we did not include any other parameters (such as the slow and fast components introduced by Smith et al. [29]).

We used the time series of movement error and the hand direction data from sets 21–70 for each participant. The data for individual participants were noisy and often included outliers; hence, we used a 3-point moving average to reduce the effects of these factors. After preprocessing the data, we applied a system identification method to estimate the parameter *k* based on the state-space model. Given 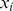 and 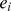, the prediction 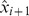 can be calculated using Eq. (1), and the squared prediction error 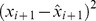 can be determined. The parameter 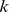 was obtained to minimize the sum of the squared prediction error from sets 21–70, by using the prediction error identification method (pem) [30] function in the System Identification Toolbox of MATLAB software (MathWorks, Natick, MA, USA).

For each trial, the reaction time (RT) was calculated as the first time point at which the hand velocity exceeded 30 mm/s (approximately 5% of the peak velocity). The movement distance (MD) was calculated as the distance from the start point to the endpoint, defined as the position at which the hand velocity decreased to <30 mm/s. The movement time (MT) was defined as the time required to reach the endpoint from the RT.

On completion of the trials, we asked the participants verbally if they thought anything unnatural had occurred during the experiment. Even if participants did not mention the rotation or delay, we subsequently asked whether they had been aware of the rotation, or the delay, or both.

### Simulation of motor learning

Using a model with identified learning rates for each condition [i.e., Eqs. (1) and (2)], we simulated the experimental task. We repeated the simulation 12 times, and calculated the average and standard deviation of the hand direction.

### Statistical analysis

One-way ANOVAs were performed to test for any significant effects of the experimental conditions on the learning index and learning rate. For RT, PV, MD, and MT analysis, the mean ± SD values were calculated for all participants across each session, and two-way ANOVAs were performed to determine the within-subject (session; repeated factor) and between-subject (condition; non-repeated factor) effects. A post hoc Tukey's test was used for multiple comparisons in any analysis where a significant main effect was observed. The statistical significance threshold was set at *P*<0.05.

## Results

**Figure 2:**
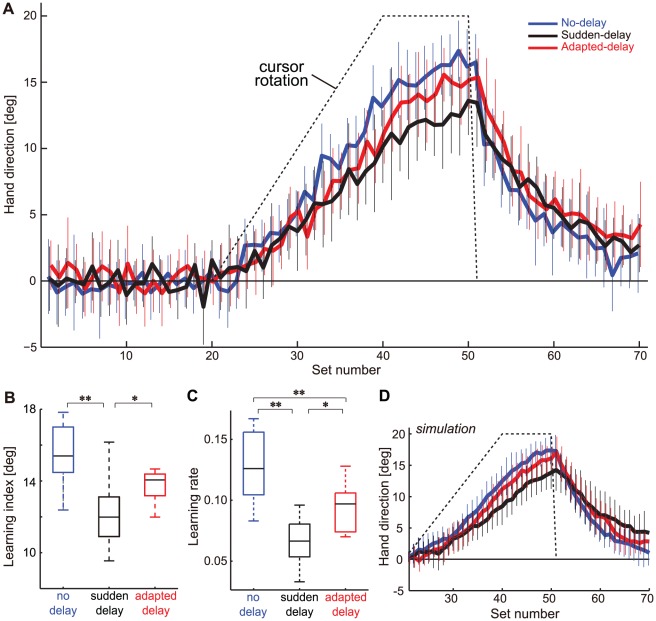
Experimental results. (**A**) Changes in hand directions for the 3 conditions (blue, no-delay; black, sudden-delay; red, adapted-delay). The broken line indicates the imposed visuomotor rotation. Values are shown as mean ± SD for all participants. (**B**) Learning indices and (**C**) learning rates for the 3 conditions. The vertical broken lines at the top and bottom of the box plots represent the maximum and minimum values, respectively. The rectangles represent the inter-quartile range (first to third quartile), and the horizontal bars in the rectangles represent the median of each variable. The asterisks indicate a significant difference (*P<0.05, **P<0.01). (**D**) The results of simulation with the identified state-space model.

Figure 2A shows the data for mean hand deviations from the target (see *Data analysis* and Fig. 1B for the definition) for the 3 consecutive sessions (baseline, learning, and washout) under each experimental condition (no-delay, sudden-delay, and adapted-delay). In the baseline session, the hand deviations were almost zero, indicating that the participants accurately moved their hands toward the targets. One-way ANOVA revealed no significant effect of experimental conditions on the hand deviations, averaged for the entire baseline session (*P* = 0.756).

We detected a significant effect of experimental conditions on the learning index (no-delay, 15.6±1.6°; sudden-delay, 12.0±2.3°; and adapted-delay, 14.2±1.6°; *P* = 0.0002). A post hoc Tukey's test revealed that the learning index of the sudden-delay condition was significantly lower than that of the no-delay condition (*P* = 0.0002), and also than that of the adapted-delay condition (*P* = 0.024) (Fig. 2B). These data indicate that the degradation of visuomotor learning caused by delayed visual feedback was alleviated by prior exposure to the delay.

We detected a significant effect of experimental conditions on the learning rate (no-delay, 0.128±0.027; sudden-delay, 0.066±0.020; and adapted-delay, 0.094±0.018; *P*<0.0001). A post hoc Tukey's test revealed significant differences in the learning rate between all experimental conditions (no-delay vs. sudden-delay, *P*<0.0001; no-delay vs. adapted-delay, *P* = 0.0031; and sudden-delay vs. adapted-delay, *P* = 0.0123; Fig. 2C).

Using a simulation based on the identified learning rate values, we successfully reproduced the experimental results (Fig. 2D). During the first half of the learning session, we observed no clear difference between the experimental conditions, because of the relatively large noise for the small error inputs. However, the difference in hand directions between experimental conditions became more apparent during the second half of the learning session, indicating the validity of the learning index to evaluate variations in motor learning performance between conditions. The simulation model also reproduced the differences in error reduction during the washout session. For direct comparison with the experimental data, we used the simulation data to calculate the learning index, and obtained the following values: no-delay, 15.7±2.1°; sudden-delay, 12.6±2.6°; and adapted-delay, 14.7±2.2°. Two-way ANOVA (condition
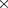
simulation/experiment) showed a significant main effect of condition (*P*<0.05). By contrast, there was no significant main effect of simulation/experiment (*P*>0.05), or significant interaction (*P*>0.05), indicating that the behavioral data were well-described by the simulation model.

To eliminate any deliberate or explicit adaptation strategy arising from conscious awareness of the visuomotor perturbation, we imposed the visuomotor rotation not abruptly, but gradually. As anticipated, none of the participants was aware of the visuomotor rotation until the end of the learning session (set 50)—all participants believed that the movement direction of the cursor indicated that of the actual hand position. Three participants thought that a clockwise visuomotor rotation had suddenly been inserted at the beginning of the washout session, because the cursor largely deviated from the target in a clockwise direction. The remaining 33 participants were unaware of the visuomotor rotation throughout the experiment. Thus, we believe that our experimental design successfully eliminated any explicit adaptation strategy arising from conscious awareness of the visuomotor perturbation, at least until the end of the learning session.

**Figure 3:**
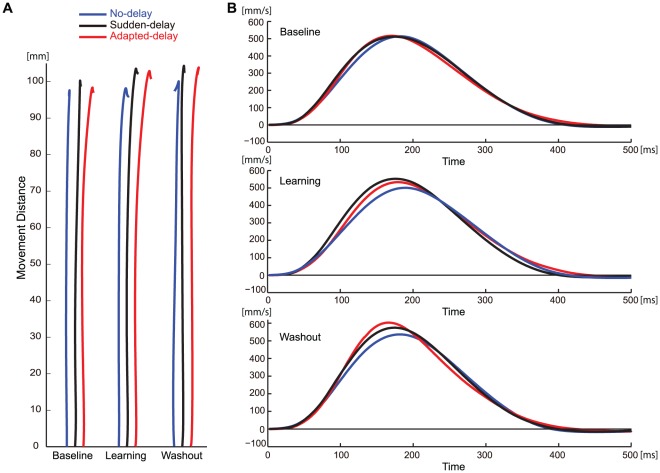
Average reaching trajectories and velocity profiles for each session under the 3 conditions. The average reaching trajectories were calculated by spatially averaging the hand position of each trial at the same time from the RT. The velocity profiles were calculated by averaging the hand velocity of each trial at the same time from the RT. (**A**) Average reaching trajectories. The movements in all directions were normalized to a single movement direction, and the spatial average was calculated. (**B**) Velocity profiles. The 40 ms in the horizontal axis indicates the time at which the hand velocity exceeded 30 mm/s.

To validate the participants' compliance to the protocol requirement stating that they should not correct their reaching movements, we calculated the average reaching trajectories and velocity profiles for the 3 conditions in each session (Fig. 3A, B). We obtained the reaching trajectories and velocity profiles by the following procedure: after normalizing all movements to a single movement direction and aligning the data with the RTs, we calculated the average of the position and velocity at every sampling time. We observed that the online corrections of reaching movement were small (Fig. 3A). Indeed, the difference in the hand movement directions calculated at the endpoint and at the peak velocity was ∼2°, even during the learning session. All of the velocity profiles were typically bell-shaped, although the PV and the time at the PV differed slightly between the experimental conditions (Fig. 3B).

**Figure 4:**
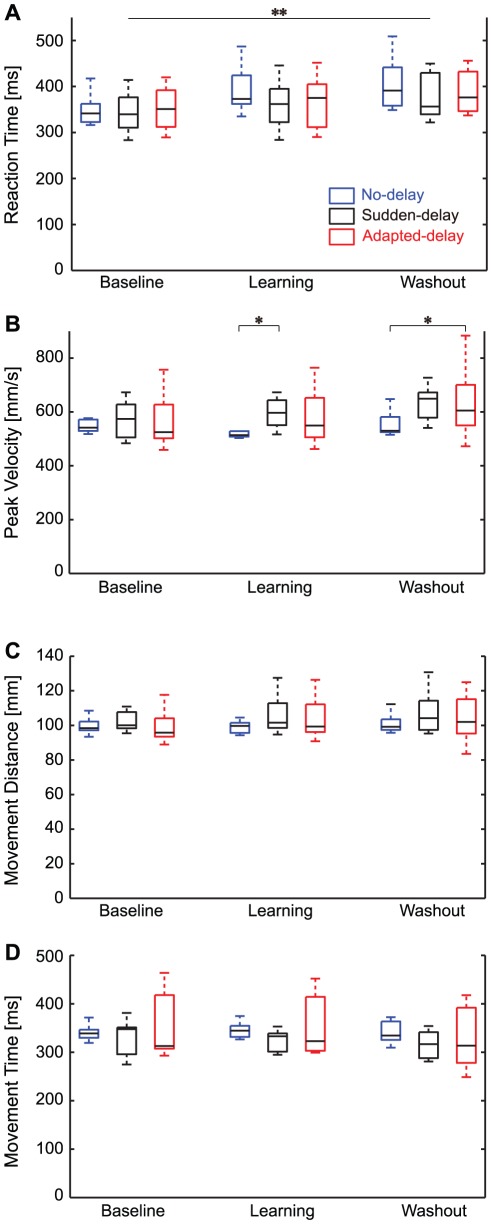
Distributions of movement parameters. (**A**) Reaction time. (**B**) Peak velocity. (**C**) Movement distance. (**D**) Movement time. The vertical broken lines at the top and bottom of the box plots represent the maximum and minimum values, respectively. The rectangles represent the inter-quartile range, and the horizontal bars in the rectangles represent the median of each variable. The asterisks indicate a significant difference (*P<0.05, **P<0.01).

The results of the RT, PV, MD, and MT measurements are summarized in Table 1 and Figure 4. With respect to RT, MD, and MT, two-way repeated-measures ANOVA revealed a significant main effect of session (*P*<0.05), but no significant main effect of condition (*P*>0.05) or interaction (*P*>0.05). Multiple comparison analysis demonstrated a significant difference in RT (*P* = 0.001) (Fig. 4A), but not in MD or MT (*P*>0.05), between the baseline and washout sessions.

**Table 1:** The statistics of RT, PV, MD, and MT.

		Sessions			*F* and *P* values of ANOVA		
Variables	Conditions	baseline	learning	washout	session	condition	interaction
RT [ms]	no-delay	346±29	392±51	406±56	*F* = 35.57	*F* = 0.67	*F* = 2.27
	sudden-delay	343±41	361±54	377±47	*P* = 0.000	*P* = 0.518	*P* = 0.07
	adapted-delay	356±44	366±53	388±46			
PV [mm/s]	no-delay	549±23	523±21	552±45	*F* = 19.26	*F* = 3.01	*F* = 4.21
	sudden-delay	572±70	600±53	631±58	*P* = 0.000	*P* = 0.063	*P* = 0.012
	adapted-delay	566±93	579±96	644±122			
MD [mm]	no-delay	101±7	100±6	101±5	*F* = 6.32	*F* = 0.99	*F* = 2.53
	sudden-delay	103±8	106±10	109±16	*P* = 0.010	*P* = 0.382	*P* = 0.077
	adapted-delay	99±9	104±12	104±13			
MT [ms]	no-delay	343±21	347±21	340±22	*F* = 10.24	*F* = 2.05	*F* = 0.39
	sudden-delay	332±26	324±20	316±28	*P* = 0.001	*P* = 0.387	*P* = 0.132
	adapted-delay	350±63	350±59	328±63			

Values represent mean ± SD calculated for all participants across each session.

We observed a significant interaction (session
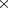
condition) on PV (*P* = 0.012). Thus, we conducted multiple comparisons between conditions for each session. In the baseline session, we detected no significant difference (*P*>0.05) between any pair of conditions. In the learning session, there was a significant difference between the no-delay and sudden-delay conditions (*P* = 0.017) (Fig. 4B) but not between any other pair. In the washout session, there was a significant difference between the no-delay and adapted-delay conditions (*P* = 0.0278) (Fig. 4B), but not between any other pair. Conditional differences were observed in PV, but not between the sudden-delay and adapted-delay conditions.

## Discussion

Delayed visual feedback was previously shown to degrade prism adaptation [3], [23]. Here, we replicated this finding using a gradual visuomotor rotation task. We observed that the learning index and learning rate degraded when a delay was artificially introduced between the hand and cursor positions (i.e., sudden-delay condition) (Fig. 2). Although it is not clear whether the underlying mechanisms are similar between prism adaptation and adaptation to a visual rotation, the learning rate obtained in our present study (0.128) was similar to those derived previously (0.091 [3] and 0.090 [23]). Our findings therefore support the validity of the state-space model [Eqs.(1) and (2)]. The de-adaptation during the washout session appeared to be slightly slower for the sudden-delay condition than for the other two conditions (Fig. 2A), as verified by the simulation result (Fig. 2D). This finding partially supports our hypothesis that the constant learning rate, which is specific to each condition, persisted throughout the experiment.

Importantly, we also demonstrated that the degradation of motor learning associated with delayed feedback was partially alleviated by prior exposure to the delay (i.e., the learning index and learning rate in the adapted-delay condition were significantly higher than those in the sudden-delay condition; Fig. 2B, C). Such learning alleviation associated with delayed visual feedback could not be explained by the changes in movement kinematics associated with conditions, because the PV, MD, and MT did not differ significantly between the 3 conditions (Fig. 4; Table 1).

We considered the possibility that the difference in learning rate between the adapted-delay and sudden-delay conditions reflected the fact that, in the learning session of the adapted-delay condition, participants only had to adapt to the visual rotation, while in the sudden-delay condition, they had to adapt to the visual rotation and the delay (similar to a dual-task design). Therefore, an alternative interpretation is that when participants first adapted to the visual delay, the difficulty of adapting to the visuomotor rotation was reduced, because more attention was assigned to the rotation adaptation alone. In this case, however, we would expect the learning rate determined by Tanaka et al. [23] to be higher in the adapted-delay condition than in the sudden-delay condition. Given that Tanaka et al. [23] did not observe such a difference, it is unlikely that the dual-task nature was solely responsible for the differences in learning rate observed between our sudden-delay and adapted-delay conditions.

It is interesting to speculate what happens during the period of exposure to visual feedback delay. Psychophysical studies previously demonstrated that, when participants were repeatedly exposed to an artificially introduced 250-ms delay between voluntary actions and sensory consequences, they perceptually combined their voluntary actions with the sensory consequences, and perceived that the delay was shortened by approximately 100 ms [4], [5]. Importantly, this finding is compatible with the theory of motor control based on the forward model. In this model, the efference copy of the motor command is processed to predict its sensory consequences. Such sensory prediction is continuously monitored and compared with the actual sensory feedback, and is used to maintain accurate predictions. Thus, perceptual binding has been considered to be caused by the formation of an appropriate temporal association between motor commands and sensory feedback (i.e., recalibration of the feedback delay in the sensorimotor loop). Based on the fact that appropriate associations between motor commands and sensory consequences are important for motor learning, we hypothesized that recalibration of the delay might alleviate the deficits of motor learning associated with delayed visual feedback. Our data confirmed the validity of our hypothesis.

Tanaka et al. [23] also examined the effects of repetitive exposure to a visual feedback delay on the learning rate of prism adaptation. They demonstrated that repetitive exposure did not induce any positive effects to motor learning, although it did shorten the subjective experience of the delay. It was concluded that the physical delay, but not the subjective delay, determined the learning rate in prism adaptation.

In contrast, we observed that the learning rate was not fully determined by differences in the physical visual feedback delay. This contradiction can be explained by 2 factors. Firstly, in our study, the cursor and target locations were continuously displayed during movement. By contrast, Tanaka et al. [23] eliminated visual feedback during movement, and allowed participants to view the target and final static position of the hand only after completion of the reach. Continuous feedback and feedback after movement were previously shown to result in considerably different outcomes of motor learning; continuous feedback facilitated visuomotor learning to a visual rotation [31]–[33]. Furthermore, according to the optimal feedback control theory [18], the CNS continuously estimates the current location of the hand, by combining sensory feedback signals with predicted signals from the forward model [19]. It uses this estimate repeatedly to correct ongoing movements [16], [17]. Thus, when continuous visual feedback is available, as in the present experiment, the CNS has the opportunity to compare sensory predictions with actual feedback at every time point during movement. This may facilitate the remapping of appropriate temporal associations between motor commands and sensory consequences during the exposure to the delay, eventually contributing to facilitation of visuomotor learning. Using a gradual visuomotor rotation task, Izawa and Shadmehr [34] demonstrated that, when the cursor was continuously displayed, participants perceived that the hand position at the end of the reach was at the cursor position. Conversely, when the cursor trajectory was not displayed, the perceived hand position at the end of the reach remained near the actual hand position. This finding indicates that continuous feedback is important for associating the hand movement with the cursor movement.

Secondly, in our study, we gradually increased the cursor rotation throughout the experiment. By contrast, Tanaka et al. [23] used a prism that necessarily imposed an abrupt visual perturbation. Motor adaptation is known to be achieved through at least 2 processes—a high-level strategic process and a low-level implicit process [21], [26]. In the previous prism experiment [23], the prism caused an abrupt perturbation, participants could not help but explicitly notice the error during the first trial of the learning session, making it difficult entirely to exclude the effect of the strategic process. By contrast, in the present study, we succeeded in eliminating the strategic process, such that none of the participants were aware of the visuomotor rotation until the end of learning session (set 50). The small change (∼25 ms) in the RT from the baseline to the learning session (Table 1) likely rules out the possibility of an explicit strategy, because Saijo et al. [35] reported a significantly longer reaction time (∼100 ms) for an abrupt visual rotation than for a gradual visual rotation. They concluded that awareness of the presence of a visual rotation or discrepancy between the hand and cursor led to an increase in the reaction time.

It is important to note that the neural bases of learning in response to abrupt and gradual perturbations are most likely distinct. Gradual introduction of either visual or force perturbations was previously shown to result in larger aftereffects [36] and enhanced retention [37], [38] following learning. The generalized pattern of adaptation differed according to whether the perturbations were introduced abruptly or gradually [39], [40]. Surprisingly, cerebellar patients were able to adapt to a force field, even when it was gradually introduced [41]. Thus, we appear to have evaluated different aspects of motor learning from those investigated by Tanaka et al. [23]. This may further explain the observed discrepancies in results between the 2 studies.

Conventionally, theories of motor learning have assumed that learning proceeds in proportion to error [42], [43]. Recently, however, an increasing number of studies have suggested that learning depends on the task relevance of error, and on the strength of the internal association between actions and their sensory feedback [28], [44]–[49]. When perturbations are either too large or very transient, the CNS regards these errors as irrelevant to our own actions, and weakly adapts to them [44], [47]–[49]. In a previous study of bimanual movements, we revealed that motor learning was affected by the strength of the association between each limb's feed-forward movement controller (i.e., internal model) and visual feedback, and that this could be manipulated by varying the location of the visual feedback [46]. In addition to these findings in the *spatial* domain, *temporal* associations between actions and sensory feedback have also been shown to be important. The attenuated adaptation to visuomotor rotation observed in rhythmic movements as compared to discrete movements was reported to be caused by an erroneous association of error information with irrelevant motor commands, which are temporally close to relevant motor commands [28], [45]. In the present study, we observed that visuomotor adaptation under delayed feedback conditions was alleviated by prior temporal binding between actions and their sensory feedback, thus further indicating the importance of appropriate temporal associations. However, this effect may be limited, because prior adaptation to delayed visual feedback only partially resolved the deficit of motor learning (Fig. 2C). In future studies, we aim to elucidate whether complete adaptation to the delay is able fully to resolve the deficit, or whether the absence of delay in visual feedback is particularly beneficial to motor learning.

In the present study, we assumed that visuomotor adaptation and recalibration of the delay are distinct and independent processes. In the same way, the *Smith Predictor* assumes that the cerebellum forms 2 separate internal models—the forward predictive model of motor apparatus and the model of feedback delay [50]. One rationale for this assumption is that the delay is a type of temporal error, and therefore should be processed differently from the well-studied spatial error. However, because our knowledge of how the delay is recalibrated in the brain is limited in computational and also in physiological terms [51], it remains unclear whether visuomotor adaptation and recalibration of the delay are indeed distinct. Given that the delay could also be regarded as a spatial error along the direction of movement (i.e., distance error), whereas the traditional spatial error made by the visual or force perturbation is a spatial error orthogonal to the direction of movement (i.e., directional error), visuomotor adaptation and recalibration of the delay may be considered as similar processes. Saunders and Knill [17] investigated online corrections for the 2 types of spatial error, by smoothly shifting visual feedback from the actual hand position along or orthogonal to the direction of movement. They demonstrated that the corrections to the 2 errors can be explained by the same online feedback mechanism. However, because the distance error was not constant during the movement, and constancy is a necessity for the occurrence of recalibration, it remains unclear whether the constant delay used in the present study is processed via the same mechanism as the traditional spatial error. Future development of an experimental paradigm to address this issue may facilitate a unified understanding of motor adaptation in terms of spatial and time domains.
